# Supplementary material for: Discovery and application of insertion-deletion (INDEL) polymorphisms for QTL mapping of early life-history traits in Atlantic salmon
Source: BMC Genomics. 2010 Mar 8;11:156. doi: 10.1186/1471-2164-11-156 (PMC2838853; doi:10.1186/1471-2164-11-156)
Supplement: Additional file 2 — Information on developed 76 locus single-run INDEL panel in Atlantic salmon. Information on fluorescence labeling, primer concentrations, PCR pooling and links to alignments, INDEL motifs and GENESCAN (Burge and Karlin 1997) predictions of genes/exons are available in html format. [file 1471-2164-11-156-S2.ZIP › Additionalfile2/snpsummary7090.html]

```
Cluster 2026 Contig 1

prev  Summary    Contig List  next
```

Size of Consensus sequence = 2752

Number of sequences = 25

Minimum redundancy = 6

Key

A gi|89834231|gb|DY694991.1|DY694991 EST\_ssal\_rgb2\_50730 ssalrgb2 mixed\_tissue Salmo salar cDNA Salmo salar cDNA clone ssal\_rgb2\_582\_073\_fwd 3', mRNA sequence  
B gi|89871852|gb|DY727975.1|DY727975 EST\_ssal\_rgb2\_83714 ssalrgb2 mixed\_tissue Salmo salar cDNA Salmo salar cDNA clone ssal\_rgb2\_637\_094\_fwd 3', mRNA sequence  
C gi|24387705|gb|CA057462.1|CA057462 ssalrgb530302 mixed\_tissue Salmo salar cDNA, mRNA sequence  
D gi|85041025|gb|DW569203.1|DW569203 EST\_ssal\_rgb2\_33622 rgb2 Salmo salar cDNA clone ssal\_rgb2\_554\_171\_fwd 3', mRNA sequence  
E gi|89835909|gb|DY695842.1|DY695842 EST\_ssal\_rgb2\_51581 ssalrgb2 mixed\_tissue Salmo salar cDNA Salmo salar cDNA clone ssal\_rgb2\_583\_188\_fwd 3', mRNA sequence  
F gi|29320682|gb|CB509456.1|CB509456 ssalnwh503109 whole Salmo salar cDNA, mRNA sequence  
G gi|24391095|gb|CA060852.1|CA060852 ssalrga515369 mixed\_tissue Salmo salar cDNA, mRNA sequence  
H gi|24384005|gb|CA053762.1|CA053762 ssalrgb502078 mixed\_tissue Salmo salar cDNA, mRNA sequence  
I gi|85047617|gb|DW575795.1|DW575795 EST\_ssal\_rgb2\_40214 rgb2 Salmo salar cDNA clone ssal\_rgb2\_565\_134\_fwd 3', mRNA sequence  
J gi|89880632|gb|DY736755.1|DY736755 EST\_ssal\_rgb2\_92494 ssalrgb2 mixed\_tissue Salmo salar cDNA Salmo salar cDNA clone ssal\_rgb2\_651\_110\_fwd 3', mRNA sequence  
K gi|24389769|gb|CA059526.1|CA059526 ssalrgb533088 mixed\_tissue Salmo salar cDNA, mRNA sequence  
L gi|24383678|gb|CA053435.1|CA053435 ssalrga516084 mixed\_tissue Salmo salar cDNA, mRNA sequence  
M gi|29322670|gb|CB511444.1|CB511444 ssalrgb547201 mixed\_tissue Salmo salar cDNA, mRNA sequence  
N gi|83640020|gb|DW006610.1|DW006610 SGP284725 Atlantic salmon Brain cDNA library Salmo salar cDNA clone HJ4-3245 5', mRNA sequence  
O gi|29322701|gb|CB511475.1|CB511475 ssalrgb530302\_rev mixed\_tissue Salmo salar cDNA, mRNA sequence  
P gi|89853568|gb|DY709691.1|DY709691 EST\_ssal\_rgb2\_65430 ssalrgb2 mixed\_tissue Salmo salar cDNA Salmo salar cDNA clone ssal\_rgb2\_605\_345\_rev 5', mRNA sequence  
Q gi|89866209|gb|DY722332.1|DY722332 EST\_ssal\_rgb2\_78071 ssalrgb2 mixed\_tissue Salmo salar cDNA Salmo salar cDNA clone ssal\_rgb2\_628\_058\_rev 5', mRNA sequence  
R gi|85041024|gb|DW569202.1|DW569202 EST\_ssal\_rgb2\_33621 rgb2 Salmo salar cDNA clone ssal\_rgb2\_554\_171\_rev 5', mRNA sequence  
S gi|85028907|gb|DW557563.1|DW557563 EST\_ssal\_rgb2\_21982 rgb2 Salmo salar cDNA clone ssal\_rgb2\_535\_287\_rev 5', mRNA sequence  
T gi|85047616|gb|DW575794.1|DW575794 EST\_ssal\_rgb2\_40213 rgb2 Salmo salar cDNA clone ssal\_rgb2\_565\_134\_rev 5', mRNA sequence  
U gi|89834229|gb|DY694990.1|DY694990 EST\_ssal\_rgb2\_50729 ssalrgb2 mixed\_tissue Salmo salar cDNA Salmo salar cDNA clone ssal\_rgb2\_582\_073\_rev 5', mRNA sequence  
V gi|29325830|gb|CB514604.1|CB514604 ssalrgb502078\_rev mixed\_tissue Salmo salar cDNA, mRNA sequence  
W gi|83639341|gb|DW005931.1|DW005931 SGP280407 Atlantic salmon Brain cDNA library Salmo salar cDNA clone HJ4-2363 5', mRNA sequence  
X gi|89880631|gb|DY736754.1|DY736754 EST\_ssal\_rgb2\_92493 ssalrgb2 mixed\_tissue Salmo salar cDNA Salmo salar cDNA clone ssal\_rgb2\_651\_110\_rev 5', mRNA sequence  
Y gi|57126846|gb|CX358287.1|CX358287 ssalrgb533088\_rev\_0 mixed\_tissue Salmo salar cDNA, mRNA sequence

2 SNPs detected

A B C D E F G H I J K L M N O P Q R S T U V W X Y  cosegregation weighted

508 . - T T - - - T T T T T T - T - . T - . . . . . .   2/2 68.00
509 . - C C - - - C C C C C C - C - . C - . . . . . .   2/2 68.00
